# Expert consensus on the diagnosis and treatment of thrombocytopenia in adult critical care patients in China

**DOI:** 10.1186/s40779-020-00244-w

**Published:** 2020-04-03

**Authors:** Jing-Chun Song, Shu-Yuan Liu, Feng Zhu, Ai-Qing Wen, Lin-Hao Ma, Wei-Qin Li, Jun Wu, on behalf of the Critical Care Medicine Committee of Chinese People’s Liberation Army (PLA); Chinese Society of Laboratory Medicine, Chinese Medical Association

**Affiliations:** 1 Department of Critical Care Medicine, the 908th Hospital of Joint Logistics Support Forces of Chinese PLA, Nanchang, 360104 China; 2 Emergency Department, the Sixth Medical Center, Chinese PLA General Hospital, Beijing, 100048 China; 3 Burns and Trauma ICU, Changhai Hospital, Naval Medical University, Shanghai, 200003 China; 4 Department of Blood Transfusion, Daping Hospital of Army Medical University, Chongqing, 400042 China; 5 Department of Emergency and Critical Care Medicine, Changzheng Hospital, Naval Medical University, Shanghai, 200003 China; 6 Surgery Intensive Care Unit, Jinling Hospital, Medical School of Nanjing University, Nanjing, 210002 China; 7 Department of Clinical Laboratory, Peking University Fourth School of Clinical Medicine, Beijing Jishuitan Hospital, Beijing, 100035 China

**Keywords:** Thrombocytopenia, Adult, Critical care, Diagnosis, Treatment, Expert consensus

## Abstract

Thrombocytopenia is a common complication of critical care patients. The rates of bleeding events and mortality are also significantly increased in critical care patients with thrombocytopenia. Therefore, the Critical Care Medicine Committee of Chinese People’s Liberation Army (PLA) worked with Chinese Society of Laboratory Medicine, Chinese Medical Association to develop this consensus to provide guidance for clinical practice. The consensus includes five sections and 27 items: the definition of thrombocytopenia, etiology and pathophysiology, diagnosis and differential diagnosis, treatment and prevention.

## Background

Platelets are blood cells directly involved in clotting and inflammatory regulation, and thrombocytopenia is a common complication of critical care patients [1]. Statistics show that the incidence of thrombocytopenia is 8.3 to 67.6% in adult critical care patients admitted to the Intensive Care Unit (ICU) and 14 to 44% during ICU stays [2]. The rates of bleeding events, blood transfusions and even mortality are also significantly increased in critical care patients with thrombocytopenia [3–5]. Therefore, the Critical Care Medicine Committee of Chinese People’s Liberation Army (PLA) worked with Chinese Society of Laboratory Medicine, Chinese Medical Association to develop this consensus to provide guidance for clinical practice.

**Fig. 1:**
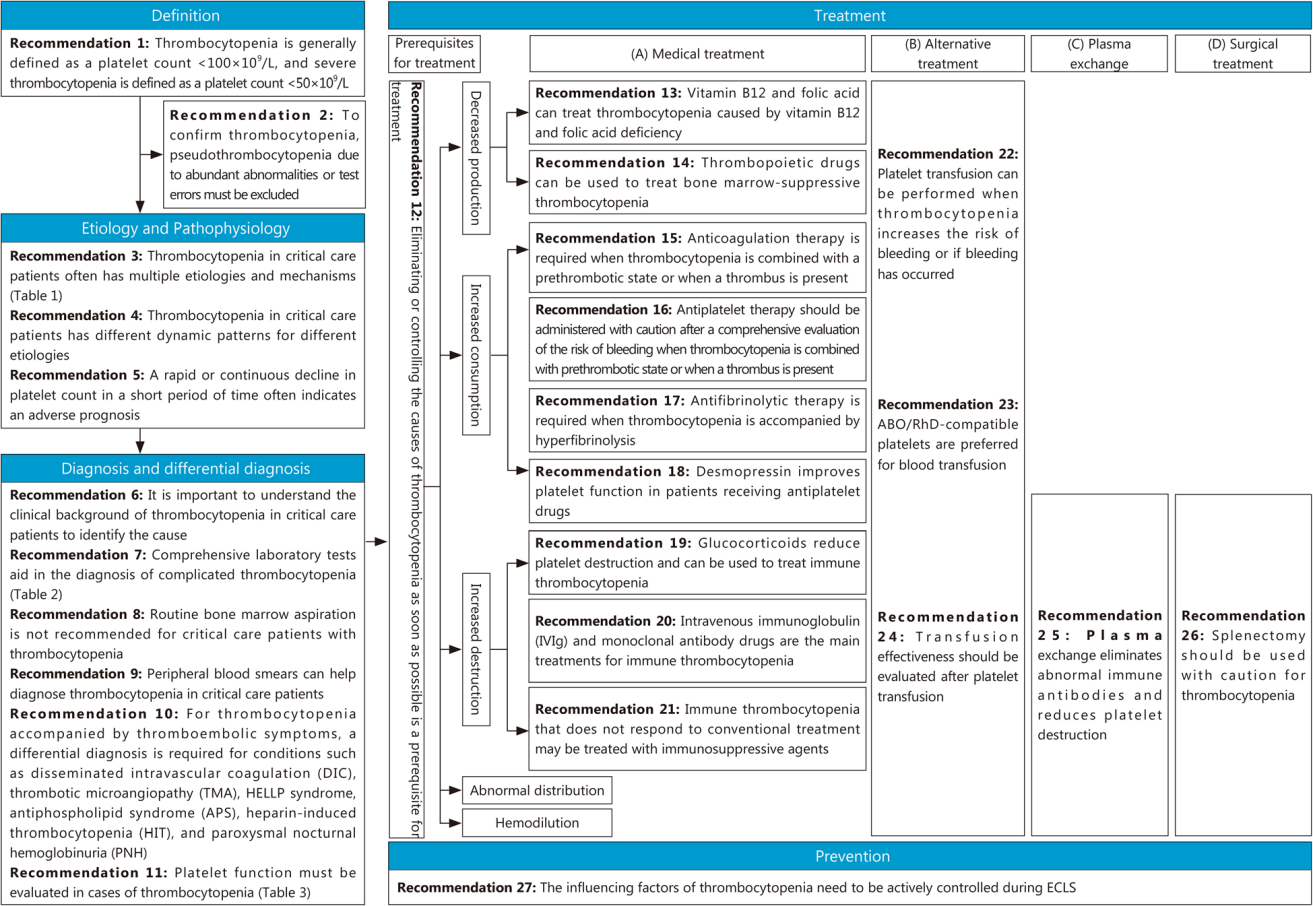
Key points of the *Expert consensus on the diagnosis and treatment of thrombocytopenia in adult critical care patients in China*

In August 2019, an expert consensus writing committee was formed by members from the Critical Care Medicine Committee of the Chinese PLA and the Chinese Society of Laboratory Medicine, Chinese Medical Association. After discussion, the committee decided that the consensus would include five sections and 27 items: the definition of thrombocytopenia, etiology and pathophysiology, diagnosis and differential diagnosis, treatment, and prevention (Fig. 1). Based on recent developments in critical care medicine, laboratory medicine, and blood transfusion medicine, the committee members met to review and discuss the content in November2019, and each speaker’s comments and recommendations were documented. After the meeting, the consensus was revised to reflect each expert’s inputs and was finalized after several conference calls and discussions.

### Definition

#### Recommendation 1: Thrombocytopenia is generally defined as a platelet count< 100 × 10^9^/L, and severe thrombocytopenia is defined as a platelet count< 50 × 10^9^/L

In healthy individuals, megakaryocytes in the bone marrow produce approximately 150 × 10^6^ platelets per day. The life span of platelets is approximately 10 days. The range of platelet counts in the normal population varies with age, sex, and ethnicity. In 2012, the former Ministry of Health of China issued *Blood Cell Analysis: Reference Ranges* as the health industry standard; it defines the normal range of platelet count as 125 to 350 × 10^9^/L. [6] Thrombocytopenia may be absolute or relative depending on the rate of decline in platelet count. In Europe and the US, absolute thrombocytopenia is usually defined as a platelet count< 150 × 10^9^/L. The normal platelet count range is usually lower in the Chinese population than in European and US populations; therefore, this consensus recommends that for Chinese adult critical care patients, absolute thrombocytopenia should be defined as a platelet count< 100 × 10^9^/L [7, 8], and relative thrombocytopenia should be defined as a platelet count decline of 30% or more from the baseline level.

#### Recommendation 2: To confirm thrombocytopenia, pseudothrombocytopenia due to sample abnormalities or test errors must be excluded

Platelet count refers to the number of platelets in a unit volume of blood. The main test methods include blood analysis, microscopic counts, and flow cytometry. Blood analysis is the main screening method as it is fast, repeatable, and accurate. The accuracy of a platelet count is subject to sample collection, transportation, storage, and testing. Poor blood flow during blood collection can easily destroy platelets. Prolonged storage or low temperature can activate platelets and lead to pseudothrombocytopenia. The diameter of normal platelets is approximately 1.5 to 3 μm; for giant platelets, which are commonly seen in Bernard-Soulier syndrome, Glanzmann thrombasthenia, and myelodysplastic syndrome (MDS), the diameter is> 7 μm.The blood analyzer usually counts cells based on cell size, making it prone to errors in identifying giant platelets and subsequently in diagnosing pseudothrombocytopenia. The anticoagulant ethylenediaminetetraacetic acid (EDTA) in the tube can chelate with calcium in the blood, leading to conformational changes in the platelet membrane glycoprotein receptorIIb/IIIa, which induces platelet aggregation [9]. The blood analyzer can mistake aggregated platelets for white or red blood cells, leading to a diagnosis of pseudothrombocytopenia. Such cases can be differentiated with blood smears and microscopic counts [10].

### Etiology and pathophysiology

#### Recommendation 3: Thrombocytopenia in critical care patients often has multiple etiologies and mechanisms

For critical care patients, thrombocytopenia can be primary or acquired. Primary thrombocytopenia is usually a result of hematological diseases, whereas acquired thrombocytopenia is more complex and may be caused by many conditions such as infection, injury, immune disorders, and nutritional disorders. Thrombocytopeniais associated with five pathophysiology mechanisms: decreased production, increased consumption, increased destruction, abnormal distribution, and hemodilution (Table 1) [7, 11, 12]. Decreased platelet production is often caused by hematological diseases, lack of nutrient substrates, or bone marrow suppression. Under normal conditions, platelets are cleared by the monocytes/macrophages of the reticuloendothelial system. In cases of immune disorders, antiplatelet autoantibodies bind to platelets and megakaryocytes, leading to increased destruction of platelets by the reticular endothelial system and subsequently to thrombocytopenia. In cases of major bleeding or extensive thrombosis, excessive consumption of platelets also leads to thrombocytopenia. Moreover, massive rehydration and blood transfusion can cause hemodilution and thrombocytopenia. Abnormal distribution is more common in spleen-related conditions and hypothermia. Under normal conditions, one-third of platelets are stored in the spleen. In the case of splenomegaly and increased spleen congestion, platelets are redistributed throughout the body, resulting in more platelets in the spleen and a decrease in circulating platelets. A condition may cause thrombocytopenia through two or more mechanisms. For example, chronic liver disease reduces platelet production due to bone marrow suppression and forms autoantibodies that lead to increased platelet destruction [13]. In addition, a patient may have two or more conditions that cause thrombocytopenia, making an accurate diagnosis more challenging [14, 15].

**Table 1:** Common causes of thrombocytopenia in critical care patients

Type		Common causes
Decreased platelet production		Severe infections (bacteria, viruses, fungi, parasites, etc.)
	Bone marrow suppression	Drugs such as valproic acid, daptomycin, linezolid, and interferon
		Poisoning, such as ethanol
		Chemotherapy drugs
		Radiation therapy
		Chronic liver disease
	Lack of nutrient substrates	Folic acid and vitamin B12 deficiency
		Pregnancy
	Hematological diseases	Leukemia, aplastic anemia (AA)
Increased platelet consumption	Bleeding	Traumatic coagulopathy, major gastrointestinal bleeding, cerebral hemorrhage
	Severe infection	Infections with bacteria (such as *Helicobacter pylori*), viruses, fungi, parasites, rickettsia, and borrelia; human immunodeficiency virus (HIV); hepatitis C; Epstein-Barr (EB) virus; mumps, measles, and rubella viruses; brucellosis; tick-borne diseases; and malaria.
	Disseminated intravascular coagulation (DIC)	Acute DIC, such as shock, infection, and leukemia; chronic DIC, such as malignant tumors and aneurysm
		Hemolysis, elevated liver enzymes, and low platelets syndrome (HELLP)
	Pregnancy-related diseases	Acute fatty liver of pregnancy (AFLP)
		Amniotic fluid embolism
		Eclampsia
	Thrombotic disease	Severe pulmonary embolism
	Extracorporeal life support (ECLS)	Extracorporeal membrane oxygenation (ECMO)
		Renal replacement therapy (RRT)
		Artificial liver support system (ALSS)
	Auxiliary circulation device	Intra-aortic balloon counterpulsation (IABP)
		Ventricularassist device (VAD)
	TMA	Thrombocytopenic purpura (TTP)
		Hemolytic uremic syndrome (HUS)
	Heat stroke	Exertional heat stroke (EHS)
		Von Willebrand disease (VWD)
	Hematological diseases	Hemophagocytic lymphohistiocytosis (HLH)
		PNH
	Autoimmune disease	APS, autoimmune hemolyticanemia (AIHA), Evan’s syndrome (AIHA + ITP)
	Hyperfibrinolytic state	Cirrhosis, metastatic prostate/ovarian tumors
Increased platelet destruction	Severe infection	Dengue
		Classic drug-dependent antiplatelet antibodies, such as quinine
	Drug-induced immune thrombocytopenia (DITP)	Hapten-induced antibodies, such as penicillin
		Drug-specific antibodies, such as tirofiban, etibeptide, and abciximab
		Drug-induced autoantibodies, such as levodopa and sulfa
		Formation of immune complex, such as HIT
	Autoimmune disease	Systemic lupus erythenlatosus (SLE), rheumatoid arthritis (RA)
Abnormal platelet distribution	Hematological diseases	Primary immune thrombocytopenia (ITP)
		Posttransfusion purpura (PTP)
	Hypersplenism	
	Low temperature	
Hemodilution	Massive rehydration or plasma transfusion	

Several studies of ICU patients with thrombocytopenia have shown that the causes of thrombocytopenia in critical care patients include (from more to less common) sepsis, DIC, dilution-induced thrombocytopenia, folic acid deficiency, malignant tumors, and drug-induced thrombocytopenia [12]. Sepsis is the most common cause of thrombocytopenia in ICU patients. Absolute thrombocytopenia is often associated with three or more mechanisms, and relative thrombocytopenia is often associated with two mechanisms.

#### Recommendation 4: Thrombocytopenia in critical care patients has different dynamic patterns for different etiologies

The dynamic patterns of thrombocytopenia are related to the characteristics of the disease. Therefore, identifying the patterns of thrombocytopenia helps to determine the cause of thrombocytopenia. Conversely, identifying the platelet count patterns of various diseases also helps to predict thrombocytopenia trends. For example, after major surgeries such as hip replacement, abdominal surgery, and cardiac surgery, platelet count usually reaches its nadir between day 1 and day 4 after operation due to tissue damage and blood loss, recovers to its preoperation level between day 5 and day 7, and peaks by day 14 after surgery [16, 17]. This pattern is related to increased thrombopoietin due to acute thrombocytopenia, and it usually takes 3 days for thrombopoietin to promote the proliferation of megakaryocytes and their division into platelets [18]. For patients with severe trauma, platelet count can decrease at 2 h after admission, and the risk of bleeding and mortality increases if the platelet count continues to decrease at the rate of 1 × 10^9^/ L/h in the following 22 h [19]. For sepsis patients, the platelet count decreases to < 150 × 10 ^9^/L within 3 days after ICU admission in 40% of patients and to < 150 × 10 ^9^/L within 5 days after ICU admission in 90% of patients, However, the platelet count can recover to its normal level within 5 days if the underlying disease is effectively controlled [20].

#### Recommendation 5: A rapid or continuous decline in platelet count over a short period often indicates an adverse prognosis

The timing, rate, and duration of thrombocytopenia and its clinical implications vary in critical care patients. A rapid or continuous decline in platelet counts over a short period often indicates acute platelet disorder and an adverse prognosis, which requires clinicians to actively identify the cause and implement interventions as early as possible [5, 21]. Past studies showed that mortality and complication rates were significantly increased in critical care patients if platelet count recovery took more than 4 days [22]. For example, Akca et al. [23] showed that the mortality rate was up to 66% in critical care patients if thrombocytopenia persisted for 14 days after ICU admission, whereas the mortality rate was only 16% if the platelet count recovered to its normal level or higher. In addition, the recovery rate helps to evaluate prognosis. Statistics show that for critical care patients with thrombocytopenia, the platelet count increases by an average of 30 × 10^9^/(L·d) in survivors and ≤ 6 × 10^9^/(L·d) in nonsurvivors [24].

### Diagnosis and differential diagnosis

#### Recommendation 6: It is important to understand the clinical background of thrombocytopenia in critical care patients to identify the cause

The collection of a complete medical history helps to determine the cause of thrombocytopenia. Clinicians should ask patients detailed questions about symptoms of bleeding or thromboembolism; infection-related symptoms (viruses, bacteria, fungi, parasites, borrelia, rickettsia); any nutritional deficiencies in the diet; liver diseases; hypersplenism; history of autoimmune diseases, such as RA and SLE; history of bariatric surgery and blood transfusion; use of medications that may cause thrombocytopenia; family history of thrombocytopenia; and the results of previous platelet tests to determine the baseline platelet count [25].

A comprehensive physical examination is required to identify signs of bleeding or embolism on the skin and any other body part. Thrombocytopenia-related bleeding mainly manifests as skin petechiae and bruising, bleeding gums, nose bleeds, menorrhagia, joint or muscle hematoma, and in severe cases, hematuria, gastrointestinal bleeding, retinal hemorrhage, and even cerebral hemorrhage. In cases of venous thrombosis, lower limb deep venous thrombosis can cause lower limb swelling; pulmonary embolism can manifest as difficulty breathing and shock; renal venous thrombosis may cause renal failure; hepatic venous thrombosis may cause Budd-Chiari syndrome; and retinal venous thrombosis may cause blindness. Arterial thrombosis may cause cerebral infarction, myocardial infarction, renal failure, and gangrene [26]. In addition, clinicians should examine any yellowing of the skin and mucous membranes; swelling of the liver, spleen, or lymph nodes; and central nervous system disorders, such as confusion, convulsions, and speech disorders. For pregnant women, placental vascular thrombosis is often associated with habitual abortion, premature birth, stillbirth, preeclampsia, and HELLP [27].

#### Recommendation 7: Comprehensive laboratory tests aid in the diagnosis of complicated thrombocytopenia

There are many causes of thrombocytopenia, and sometimes, the cause may be misdiagnosed at the initial visit. Therefore, the following screening tests are recommended for patients with thrombocytopenia of an unknown cause (Table 2) [13, 14]. If routine screenings cannot determine the cause, additional tests may be performed based on the patient’s medical history and clinical manifestations. For example, patients who have undergone platelet transfusion in the past 3 weeks can be tested for platelet-related antibodies to exclude PTP. Patients with recurrent arteriovenous embolism can be tested for anticardiolipin-antibodies (aCL), lupus anticoagulant (LAC), and β_2_ glycoprotein-I (β_2_ GPI) antibodies [28]. Patients with thrombocytopenia, microangiopathic hemolysis, neurological symptoms, fever, and renal impairment may be tested for von Willebr and factor (vWF)- cleaving protease(ADAMTS13) activity and ADAMTS13 inhibitors [29]. Patients with clinically suspected hemophilia but normal factor VIII and IX activities may be tested for vWF antigen and activity to rule out VWD [30]. Patients with heparin exposure in the past 3 months and 4Ts score ≥ 3 may be tested for HIT antibodies [31]. The 4Ts score is mainly used during initial HIT diagnosis and is the sum of four subscores, including the number of thrombocytopenia features, the duration of thrombocytopenia, the type of thrombosis, and other causes of thrombocytopenia. It is used to determine the likelihood of HIT: ≤ 3, low; 4 to 5, possible; 6 to 8, probable.

**Table 2:** Routine screening for thrombocytopenia

Item	Diagnosis
Blood test; C-reactive protein (CRP); procalcitonin (PCT)	Severe infection; hematological disease
Prothrombin time (PT), activated partial thromboplastin time (APTT), thrombin time (TT), fibrinogen (Fib), D-dimer (DD), fibrindegradation products (FDP)	DIC; the cause should be determined
Alanine aminotransferase (ALT), aspartate aminotransferase(AST), creatinine (Cr), total bilirubin (TBil)	Thrombocytopenia due to liver and renal impairment
Lactic acid dehydrogenase (LDH)	Hemolytic anemia; paroxysmal nocturnal hemoglobinuria
Vitamin B12, folic acid	Malnutrition-associated thrombocytopenia
EBvirus, cytomegalovirus (CMV), hepatitis B virus (HBV), hepatitis C virus (HCV), HIV	Viral infection
Antinuclear antibodies (ANA), rheumatoid factor (RF)	Autoimmune diseases such as SLE, Sjogren’s syndrome, and RA

#### Recommendation 8: Routine bone marrow aspiration is not recommended for critical care patients with thrombocytopenia

For ICU patients, thrombocytopenia is usually caused by sepsis, DIC, malnutrition, and drugs. Therefore, routine bone marrow aspiration is not recommended. However, bone marrow aspiration may be performed in cases of unknown cause and the presence of abnormalities of other blood cells [32]. Thiolliere et al [14]analyzed the results of bone marrow aspiration in 255 ICU patients and found that for patients with absolute thrombocytopenia, bone marrow aspiration showed (from more to less common) sepsis-related bone marrow features characterized by megakaryocyte depletion,normal bone marrow, folic acid- and vitamin B12 deficiency-related changes, megaloblastosis, and hematological tumors. HLH should be considered in cases of thrombocytopenia with fever (temperature > 38.5 °C for > 7 d), splenomegaly, hemoglobin < 90 g/L or neutrophil count < 1.0 × 10^9^/L, hypertriglyceridemia, hypofibrinogenemia, elevated serum ferritin, decreased natural killer (NK) cell activity, and elevated soluble CD25; and bone marrow aspiration should be performed to identify hemophagocytes to confirm the diagnosis [33].

#### Recommendation 9: Peripheral blood smears can help diagnose thrombocytopenia in critical care patients

Peripheral blood smears help to analyze the amount, size, and morphology of platelets; determine excessive platelet destruction or consumption [34]; identify pseudothrombocytopenia [8, 9]; and differentiate acute febrile diseases such as sepsis, dengue, and Leptospira infection through granulocytic changes [35]. On peripheral blood smear, aberrant morphology of red blood cells or red blood cell fragments is a characteristic feature of TMA [36]. Moreover, red blood cells that are swollen or varied in size indicates folic acid or vitamin B12 deficiency. Deformed spherical, target-shaped, crescent, or angular red blood cells indicate hemolytic diseases such as DIC and AIHA. Large platelets (> 4 μm) are seen in Bernard-Soulier syndrome, Glanzmann thrombasthenia, myeloid leukemia, and ITP. Small platelets (< 1.5 μm) are seen inAA. Peripheral blood smear should be performed at the same time as bone marrow aspiration [37].

#### Recommendation 10: For thrombocytopenia accompanied by thromboembolic symptoms, differential diagnosis is required for conditions such as DIC, TMA, HELLP syndrome, APS, HIT, and PNH

Thrombocytopenia usually manifests as bleeding. In the case of thromboembolism or hemolysis, differential diagnosis is required for conditions such as DIC, TMA, HELLP, APS, HIT, and PNH [38]. In the case of DIC, many causes can lead to local microvascular injury, triggering extensive activation of the coagulation system and organ dysfunction. Sepsis-induced DIC is the most common type of DIC, and its incidence is 150 times that of TMA [39]. The main laboratory features of sepsis-induced DIC include thrombocytopenia, increased consumption of coagulation factors, and elevated fibrin markers.

TMA is usually secondary to specific conditions, such as malignant tumors, infection, collagenous disease, and pregnancy. TMA includes TTP, atypical HUS, and Shiga toxin-associated HUS. Despite having different mechanisms, these conditions share the common feature of microangiopathic hemolytic anemia, especially hemolytic anemia, thrombocytopenia, and organ failure [40].

Both DIC and TTP can cause microvascular thrombosis, but DIC mainly causes postcapillary small venous thrombosis due to activation of the coagulation system, whereas TTP mainly causes postcapillary small artery thrombosis due to platelet and vWF microaggregation [41]. Acquired TTP is caused by ADAMTS-13 autoimmune responses; as a result, ADAMTS-13 activity is typically < 10% in TTP and > 30% in DIC and other types of TMA, which is helpful for differential diagnosis [42].

Atypical HUS is characterized by vascular endothelial injury due to the excessive activation of complements, as well as platelet activation and hemolytic response [43]. Hemolysis can lead to the excessive release of red blood cell injury-related molecules such as free heme, which then induces the production of neutrophil extracellular traps (NETs), activates inflammatory-coagulant interactions, and leads to extensive thrombosis [44, 45]. Shiga toxin-associated HUS is more common than atypical HUS. Shiga toxin-associated HUS is caused by Shiga toxin-producing *Escherichia coli*, especially O157: H7 or O104: H4, which causes gastrointestinal infection, produces toxins that cause endothelial cell damage, induces complement deposition in endothelial cells, and interferes with complement activity [46]. Upon diagnosis, the preferred treatment for Shiga toxin-associated HUS is supportive care rather than antibiotics.

HELLP, a serious complication of pregnancy-associated preeclampsia, is characterized by impaired ADAMTS-13 activity, excessive release of vWF multimers, the production of activated vWF, and subsequent microvascular platelet thrombosis [47]. Thrombocytopenia, microangiopathic anemia, and liver damage are the main clinical manifestations of HELLP. Timely delivery is the standard of care for HELLP. Acquired TTP should be considered if symptoms continue to worsen after delivery [48].

HIT is a condition induced by platelet-activating antibodies during treatment with heparin. It mainly manifests as thrombocytopenia, as well as venous and arterial thrombosis, and even death [49]. Literature review shows that the incidence of HIT is approximately 0.1 to 5.0%, and the incidence is higher with unfractionated heparin use than with low-molecular-weight heparin use [50]. HIT is mainly caused by platelet factor 4 (PF4)-heparin complex, which stimulates immune cells to produce PF4-heparin complex antibodies (i.e., HIT antibodies), which in turn leads to persistent platelet activation, activation of the coagulation pathway, platelet thrombosis, and fibrin thrombus [51]. HIT is diagnosed if the 4Ts score is ≥4 in the presence of positive IgG-specific HIT antibodies [52]. HIT may be misdiagnosed in the presence of sepsis because both conditions may manifest as leukocytosis and ischemic limb necrosis. However, HIT often causes lower limb venous thrombosis, whereas DIC is more likely to cause symmetrical peripheral gangrenes [53].

APS is an autoimmune disease characterized by recurrent arteriovenous thrombosis, habitual abortion, cytopenia, and persistently positive (medium to high titers) antiphospholipid antibodies. It is most common in young women. Habitual abortion and intrauterine stillbirth are the main clinical manifestations in female APS patients [54]. Thrombosis can occur in any artery or vein, regardless of its size. A small number of patients may have multiple incidents of thrombosis within a week and subsequently develop acatastrophic vascular occlusion called catastrophic antiphospholipid syndrome (CAPS). APS is confirmed with routine tests that show thrombocytopenia, neutropenia, hemolytic anemia, and positive serum antiphospholipid antibodies (aCL, LAC, β_2_ GPI antibodies) [55].

PNH is an acquired hemolytic disease resulting from defects in red blood cell membrane due to hematopoietic stem cell mutations. The main clinical manifestations are intravascular hemolytic anemia, cytopenia due to bone marrow failure, thrombosis, and smooth muscle dysfunction. Thrombosis often occurs in the hepatic vein, followed by mesenteric vein, cerebral vein, and lower lime deep vein, and arterial thrombosis is rare [56]. Typical cases show characteristic paroxysmal nocturnal hemoglobinuria. Flow cytometry is the gold standard for the diagnosis of PNH.

#### Recommendation 11: Platelet function must be evaluated in case of thrombocytopenia

Platelet count directly affects platelet function. However, platelet function and platelet count are not necessarily in sync [57] in critical care patients due to different causes and stages and concomitant antiplatelet therapy in some patients [58, 59]. Certain herbals or foods may inhibit platelet function, such as ginkgo, garlic, ginger, angelica, feverfew, ginseng, hawthorn, turmeric, and rographis, and dogwood [60]. Moreover, the early stage of sepsis is characterized by significantly enhanced platelet adhesion and aggregation. Even in the case of thrombocytopenia, platelet may still be hyperfunctional in sepsis patients [60], followed by decreased platelet aggregation along with excessive platelet consumption [61–63]. Trauma patients, especially those with brain injury, may experience decreased platelet aggregation even if platelet count is still in the normal range [64–66]. Trauma patients with prolonged use of oral antiplatelet drugs are particularly prone to adverse prognosis due to platelet dysfunction [67, 68]. For acute myeloid leukemia (AML) patients with thrombocytopenia, platelet aggregation and activation tests are superior to platelet count in predicting the risk of bleeding [69]. Therefore, platelet function should be evaluated in case of thrombocytopenia.

Conventional platelet function tests analyze platelet adhesion, aggregation, and release during hemostasis [70, 71].Viscoelasticity-based tests(thromboelastography, coagulation and platelet function analyzer) can all comprehensively reflect the overall function of coagulation factors, fibrinogen, and platelets; and are used to guide platelet replacement therapy and antiplatelet therapy (Table 3) [72, 73]. Viscoelasticity-based tests such as thromboelastography and coagulation and platelet function analyzer indicate the contribution of platelets during coagulation. The results cannot be explained in the same manner as those of conventional platelet function tests because of different principles [74, 75]. For platelet function, the coagulation and platelet function analyzer is less affected by fibrinogen interference and is thus more accurate than thromboelastography [76, 77]. Light transmission aggregometry (LTA) and platelet function analyzer 100 (PFA-100) are susceptible to the effect of thrombocytopenia (< 100 × 10^9^/L) and are thus not recommended for routine platelet function monitoring in patients with thrombocytopenia [78].

**Table 3:** Platelet function tests for patients with thrombocytopenia

Experiment name	Experimental principle	Clinical significance	Limitations
Thrombelastography	Based on blood viscoelasticity, the device activates coagulation through needle rotation, thus simulating coagulation in vitro	1. It comprehensively reflect the function of coagulation factors, platelets, and fibrinolysis system 2. Detect residual heparin and guide heparin dosage 3. The graph can be used to evaluate the efficacy of thromboxane A2 (TXA2 in) inhibitors and P2Y12 receptor inhibitors.	Long test time
Coagulation and platelet function analyzer	Based on blood viscoelasticity, the device observes vertical needle vibration to simulate coagulation in vitro	It comprehensively reflect the function of coagulation factors and platelets and is particularly accurate for evaluating platelet function	
Whole-blood platelet aggregation rate	Changes in platelet aggregation electrode impedance caused by different stimuli	1. Posttrauma or postoperative platelet function evaluation, and platelet count measurement 2. Evaluation of the efficacy of antiplatelet drugs (TXA2 inhibitors, P2Y12 receptor inhibitors)	1. The result may be affected when platelet count < 27 × 10^9^/L 2. Test must be performed within 10 min of sample collection
VerifyNow platelet function analysis	Cassette detection based on changes in light signals during platelet aggregation	1. Posttrauma or postoperative platelet function evaluation to guide platelet transfusion 2. Evaluation of the efficacy of antiplatelet drugs (TXA2 inhibitors, P2Y12 receptor inhibitors, glycoproteins IIb/IIIa receptor inhibitors)	1. Not recommended for hereditary platelet dysfunction 2. No established data on the effect of thrombocytopenia on the result
Flow cytometry	Detection of fluorescent-labeled antibody and cell size via light scattering	1. To diagnose defects in platelet surface glycoproteins or platelet secretion 2. To detect platelet-associated antibodies	Expensive

### Treatment

#### Recommendation 12: Eliminating or controlling the causes of thrombocytopenia as soon as possible is a prerequisite for treatment

There are many causes of thrombocytopenia. To treat thrombocytopenia, clinicians must first eliminate or control the factors inducing thrombocytopenia as soon as possible. Discontinuation of drugs that cause thrombocytopenia is a first-line treatment for DITP [79]. Effective infection management is the prerequisite for treating sepsis-associated thrombocytopenia [80]. To treat thrombocytopenia caused by active bleeding, it is first necessary to effectively stop the bleeding [81]. For thrombocytopenia caused by immune diseases, the excessive immune response must be effectively controlled [82]. For ECLS-associated thrombocytopenia, the treatment plan should be adjusted in a timely manner to prevent excessive platelet consumption [83].

### Medical treatment

#### Recommendation 13: Vitamin B12 and folic acid can treat thrombocytopenia caused by vitamin B12 and folic acid deficiency

Vitamin B12 and folic acid are important coenzymes for the synthesis of deoxyribonucleic acid (DNA). Critical care patients require more vitamin B12 and folic acid, which may lead to relative vitamin B12 and folic acid deficiency and subsequently tothrombocytopenia, megaloblastic anemia, and hyperhomocysteinemia [84]. Studies have shown that vitamin B12 or folic acid deficiency is associated with the severity of patient condition [85]. Folic acid deficiency may also occur during RRT [86]. Thiolliere et al [14] showed that folic acid and vitamin B12 deficiency-related changes are common in bone marrow aspiration samples from ICU patients. Vitamin B12 deficiency may be accompanied by neurological symptoms. Folic acid deficiency may be accompanied by malignant anemia and mental disorders. Blood tests may indicate pancytopenia, and bone marrow aspiration may show myelodysplasia. Upon diagnosis, vitamin B12 and folic acid supplements can relieve symptoms.

#### Recommendation 14: Thrombopoietic drugs can be used to treat bone marrow suppressive thrombocytopenia

Thrombopoietin (TPO), a thrombopoietin receptor agonist, promotes megakaryocyte division into platelets and platelet release. It is mainly used to treat postchemotherapy thrombocytopenia, ITP, AA, and hepatitis-associated thrombocytopenia [87–91]. Some studies showed that for sepsis-associated thrombocytopenia, TPO treatment improved platelet count and reduced platelet transfusion [92]; however, other studies showed that blocking thrombopoietin reduced organ damage associated with sepsis [93]. Therefore, the role of TPO is still controversial in terms of improving the prognosis of sepsis.

Interleukin-11 (IL-11) is a cytokine produced by stromal cells and mesenchymal cells in the hematopoietic microenvironment. IL-11 binds to the specific receptor IL-11Rα on the cell surface to stimulate proliferation of hematopoietic stem cells and megakaryocytic progenitor cells, induce differentiation and maturation of megakaryocytes, and promote platelet production [94, 95]. IL-11 has been approved for chemotherapy-related thrombocytopenia, leukemia, AA, and ITP [8, 96–98].

#### Recommendation 15: Anticoagulation therapy is required when thrombocytopenia is combined with a prethrombotic state or a thrombus is already formed

Prethrombotic state is a pathological state with high risk for thrombosis. The mechanisms include endothelial cell injury, elevated platelets and coagulation factors or their activity, decreased anticoagulants and fibrinolytic components or their activity, and slow blood flow. If thrombocytopenia is combined with prethrombotic state, anticoagulation therapy is required in order to prevent thrombosis. Immune dysfunction or severe infection often requires anticoagulation therapy as the condition may lead to multiple thrombosis and platelet depletion. Common anticoagulants include unfractionated heparin, low-molecular-weight heparin, argatroban, and bivalirudin. The choice of drugs depends on the mechanism and characteristics of the condition [99]. Anticoagulationtherapy is contraindicated for active bleeding.

Anticoagulation therapy may beinitiated in APS patients with recurrent lower limb deep venous thrombosis, pulmonary embolism, persistently high antibody titers, or hypercoagulable state. For recurrent venous thrombosis, unfractionated heparin or low-molecular-weight heparin (anti-Fxa, 0.3 to 0.7 U/mL) may be used in combination with warfarin for 3 to 5 days, followed by warfarin alone for 12 months to maintain prothrombin time/international normalized ratio (PT-INR) at 2.5. For recurrent arterial thrombosis, warfarin may be used for 12 months to maintain PT-INR at 3.0 [100, 101].

For patients with highly suspected or confirmed HIT, heparin should be discontinued and replaced with nonheparin anticoagulants, such as argatroban or bivalirudin [31, 51]. Argatroban is metabolized in the liver and can lead to significantly prolonged TT. The recommended starting dose of argatroban is 0.2 to 0.5 μg/kg/min iv; the dose may be adjusted for patients with liver failure. The active anticoagulation ingredient of bivalirudin is hirudin-derived fragment, which is reversible and fast-acting and has a half-life of 25 to 30 min. The initial dose is 0.05 mg/kg/h. The anticoagulation treatment target is APTT elongation of 1.5 to 3.0 times the baseline level (≤ 100 s). After dose adjustment, APTT is monitored every 4 h and then every day once the target has been met for at least 2consecutive times [102]. After the condition is stabilized, the patient may be switched to oral anticoagulants once the platelet count is ≥100 × 109 L or has returned to baseline level. If warfarin is used, it must be used with intravenous anticoagulants for 5 days and then alone once the INR target has been met. If novel oral anticoagulants (NOACs) such as rivaroxaban are used, they may be started in 2 h after the discontinuation of intravenous anticoagulants [103].

Patients with sepsis-associated DIC are prone to extensive microthrombosis load and multiorgan dysfunction due to the upregulation of the procoagulant mechanism, an impaired anticoagulant mechanism, and the inhibition of fibrinolysis. Anticoagulation therapy protects the vascular endothelium, reduces platelet and coagulant consumption, reduces the thrombus load, and protects organ function [104]. Studies have shown that heparin anticoagulants reduce the mortality of sepsis [105, 106]. For sepsis patients, anticoagulation therapy should be administered in cases of persistent decline in platelet count, persistent elongation of coagulation time, organ dysfunction due to microthrombosis, and when required to manage thrombotic events duringthe hypercoagulable stage of DIC [107]. If the patient is in a hypocoagulable state and at risk for bleeding, anticoagulation therapy may be providedwhile replenishing the patient’s coagulation substrates [108]. Randomized controlled trials (RCTs) have shown that recombinant activated protein C, tissue factor pathway inhibitor (TFPI), and thrombomodulin (TM) have no effect in improving the prognosis of sepsis [109–111].

For patients with heat stroke, significantly elevated core temperature leads to extensive endothelial cell injury, full activation of the coagulation system, rapid platelet consumption, and even DIC. Heatstroke-induced DIC also requires anticoagulation therapy while replenishing coagulation substrates. Unfractionated heparin is the preferred treatment [112].

#### Recommendation 16: Antiplatelet therapy should be administered with caution after a comprehensive evaluation of the risk of bleeding when thrombocytopenia is combined with a prethrombotic state or a thrombus is already formed

For patients with thrombocytopenia, compensatory platelet hyperaggregation may increase the risk of platelet thrombosis [113]. Studies have shown that after coronary bypass grafting, the incidence of thrombocytopenia is 71.5%, and the risk of delayed ischemic stroke (≥ 2 days after operation) increases by 12% for every 30 × 10^9^/L decline in platelet count [114]. However, no guidelines have been established for antiplatelet therapy in patients with thrombocytopenia. Myles et al. [115] showed that for coronary bypass grafting, preoperative discontinuation of aspirin has no effect on postoperative bleeding or thrombosis. On the other hand, Saw et al. [116] showed that after coronary bypass grafting, ticagrel or combined with aspirin reduces embolic complications of the graft. Therefore, accurate evaluation of platelet function and coagulation state in patients with thrombocytopenia is a prerequisite for antiplatelet therapy planning. Antiplatelet therapy may be started in cases of thrombocytopenia combined with a high risk of thrombosis [117]. After the start of antiplatelet therapy, the treatment response should be monitored to evaluate efficacy and the risk of bleeding.

For patients with acute coronary syndrome (ACS), the incidence of thrombocytopenia is as high as 13%, especially in elderly patients with diabetes or heart and kidney dysfunction [118]. For ACS patients, antiplatelet therapy in the presence of thrombocytopenia increases the risk of bleeding; therefore, care must be taken during antiplatelet therapy to avoid concomitant nonsteroidal anti-inflammatory drugs and glycoprotein IIb/IIIa receptor antagonists. Moreover, aspirin should be started at a low dose, triple-antiplatelet therapy should be avoided in patients with prolonged anticoagulation therapy, concomitant proton pump inhibitors may be provided, and drug-coated stents should be used whenever possible [119]. Patients who must undergo percutaneous coronary interventions may be given aspirin combined with clopidogrel as antiplatelet therapy for 1 month, followed by clopidogrel alone when the platelet count reaches 50 to 100 × 10^9^/L with no active bleeding. Patients who have not undergone percutaneous coronary interventions may be given clopidogrel alone as antiplatelet therapy. All antiplatelet drugs should be discontinued and percutaneous coronary intervention avoided if the platelet count is < 50 × 10^9^/L or if active bleeding occurs.

#### Recommendation 17: Antifibrinolytic therapy is required when thrombocytopenia is accompanied by hyperfibrinolysis

Thrombocytopenia caused by trauma-induced bleeding, severe liver disease, acute promyelocytic leukemia, or snake bite is often accompanied by hyperfibrinolysis [120]. Bleeding due to severe trauma can lead to thrombocytopenia; meanwhile, excessive release of protein C inhibits the activity of plasminogen activator inhibitior-1 (PAI-1), resulting in relatively enhanced tissue-type plasminogen activator (t-PA) activity and hyperfibrinolysis, which requires antifibrinolytic therapy. In the case of a propensity towards major bleeding after severe trauma, tranexamic acid (1 g iv over 10 min) should be administered as soon as possible, followed by 1 g iv over 8 h [121, 122]. For VWD patients with thrombocytopenia, antifibrinolytic therapy may be administered as an adjuvant treatment in cases of oral mucosal bleeding or excessive menstrual bleeding [123, 124].

#### Recommendation 18: Desmopressin improves platelet function in patients receiving antiplatelet drugs

1-deamino-8-D-arginine vasopressin (DDAVP) promotes the release of vWF and factor VII from endothelial cells, promotes the expression of platelet membrane glycoproteins and enhances platelet adhesion and aggregation. It is the preferred treatment for VWD-related bleeding [125–127]. Clinical studies have shown that DDAVP improves postoperative platelet function and bleeding time in uremic patients [128], improves platelet aggregation in patients taking aspirin and/orclopidogrel [129–131], and reduces postoperative bleeding in cardiac patients taking aspirin [132, 133], with very low incidences of cardiovascular and cerebral vascular thrombosis complications [134]. DDAVP (0.4 μ/kg iv over 30 min) may be given in cases of cerebral hemorrhage in patients receiving antiplatelet drugs or in cases of injury in VWD patients [135, 136].

#### Recommendation 19: Glucocorticoids reduce platelet destruction and can be used to treat immune thrombocytopenia

Glucocorticoids inhibit the production of autoantibody IgG, stabilize platelets and the endothelial cell membrane, reduce the destruction of platelets and red blood cells, and stimulate bone marrow hematopoiesis. They are the first-line treatment for ITP and the main treatment for TTP and CAPS [137–139].

Glucocorticoid regimens vary according to the specific cause of thrombocytopenia. For ITP, the general recommendation is for oral glucocorticoids, such as prednisone 1 mg/kg/day, which can be tapered to 5 to 10 mg/day for 3 to 6 months once the patient’s condition has stabilized. During tapering, the minimum maintenance dose may be given in cases of thrombocytopenia. The prednisone dose should be quickly reduced and the drug should be withdrawn in the case of a lack of response after 4 weeks of treatment. Moreover, oral dexamethasone may be given at 40 mg/day for 4 days and may be repeated in 2 weeks in the case of a lack of response [89, 140].

Studies have shown that for TTP, high-dose glucocorticoids (methylprednisolone 10 mg/kg/day) is more effective than low-dose glucocorticoids (methylprednisolone 1 mg/kg/day). Therefore, for TTP, methylprednisolone 1 g/day iv over 2 h for 3 days immediately after plasma exchange is recommended [141]. No empirical evidence is available for the tapering regimen, which is generally based on the platelet count and ADAMTS13 level. Except under special circumstances, the patient may be switched to prednisone 1 mg/kg/day, followed by tapering once the patient’s condition has stabilized [142]. Dexamethasone 40 mg/day for 4 days may be used to treat relapsed or refractory ITP or as an alternative to first-line treatment [143]. High-dose dexamethasone may increase the platelet count in a shorter time, but whether its overall response is superior to that of methylprednisolone is inconclusive [144]. Despite the lack of empirical evidence, high-dose glucocorticoids are recommended for the treatment of CAPS [145]. Septic shock patients often have thrombocytopenia and may receive hydrocortisone 200 mg/day iv in the case of hemodynamic instability after sufficient rehydration and vasoactive treatment [146].

#### Recommendation 20: Intravenous immunoglobulin (IVIg) and monoclonal antibody drugs are the main treatments for immune thrombocytopenia

IVIg was used as a first-line treatment for ITP as long agoin 1981, with a response rate of 70 to 80% for ITP. γ-globulin competitively inhibits binding between antigen-presenting cells and T cells, blocks activated Fcγ receptors, upregulates the inhibitory receptor Fcγ RIIB, inhibits the complement cascade, and neutralizes pathological autoantibodies and pathogenic cytokines, thus playing a role in regulating immune balance [147]. The dose of IVIg is usually 400 mg/(kg·d) for 5 days or 1 g/(kg·d) for 1 to 2 days. IVIg is faster-acting than glucocorticoids for the treatment of ITP and usually takes effect in 24 to 48 h; therefore, it is often used to treat ITP with major bleeding, ITP patients who require emergency invasive surgery (preoperative preparation), and refractory ITP [148].

IVIg combined with plasma exchange is considered a powerful treatment for refractory TTP and CAPS [149, 150]. Moreover, IVIg may be administered to treat DITP or PTP with severe thrombocytopenia (platelet count < 5 × 10^9^/L) or life-threatening bleeding [151]. High-dose IVIg may be effective for HIT that does not respond to conventional anticoagulation therapy [146, 152, 153]. Currently, IVIg is not recommended for the treatment of septic shock associated with thrombocytopenia [154].

Rituximab, a human-mouse chimeric monoclonal anti-CD20 antibody, clears B lymphocytes from the blood, lymph nodes, and bone marrow. The standard dose is 375 mg/m^2^ once a week for 4 weeks. Moreover, rituximab 100 mg once a week for 4 weeks is equally effective but takes longer to take effect [89]. Rituximab can be used as a first-line treatment for severe or recurrent TTP and as a second-line treatment for ITP. Caplacizumab is a potent and selective bivalent anti-vWF nanobody that has been approved in Europe for acquired TTP. It blocks the interaction between ultra large vWF multimers (ULvWF) and platelets and can be used as a first-line treatment for severe TTP [142].

#### Recommendation 21: Immune thrombocytopenia that does not respond to conventional treatment may be treated with immunosuppressive agents

For most cases of immune thrombocytopenia, immunosuppressive agents can be used as a second-line treatment for APS, AIHA, ITP, and TTP that does not respond to conventional treatment [28, 89, 142, 155]. Moreover, immunosuppressive agents can be used as a first-line treatment for immune diseases that manifest mainly as pancytopenia, such as HLH [156]. Common immunosuppressive agents include vincristine (VCR), cyclosporine A, cyclophosphamide, chloroquine, azathioprine, triptolide, and danazol. Because of their significant side effects, immunosuppressive agents are usually used in patients who do not respond to conventional treatment, and the treatment regimen must be individualized. Immunosuppressive agents can reduce the dose of glucocorticoids in combination therapy.

### Alternative treatment

#### Recommendation 22: Platelet transfusion can be performed when thrombocytopenia increases the risk of bleeding or if bleeding has occurred

Three platelet products are commonly used in China. Platelets prepared from 200 ml of whole blood are referred to as 1 unit of platelet concentrate. The concentration and purity of platelet concentrate are high, with≥2.0 × 10^10^ platelets per unit. Generally, multiple bags are required. Two or more bags of platelet concentrate are pooled into one bag to prepare pooled platelet concentrate, with ≥2.0 × 10^10^ platelets per pooled unit. Platelets collected with anapheresis machine from the circulating blood sample of a single donor are called apheresis platelets or hemapheresis platelets. The purity of this product is high, with≥2.5 × 10^11^ platelets per unit, and it is superior to platelet concentrate for reducing the risk of alloimmune responses [157].

Platelet transfusion is used to prevent and treat bleeding in patients with thrombocytopenia or platelet dysfunction. It may be delivered as a preventive transfusion (in cases of thrombocytopenia or platelet dysfunction with no bleeding) or therapeutic transfusion (in cases of thrombocytopenia or platelet dysfunction with signs of bleeding) [158]. Preventive transfusion is contraindicated in patients with PTP, TTP, HUS, or HIT with no life-threatening bleeding [32]. Before platelet transfusion, clinicians should evaluate the risk of bleeding, platelet count and platelet function, and any scheduled invasive treatment [159].

For nonbleeding patients in stable condition, platelet transfusion is recommended if the platelet count is ≤10 × 10^9^/L; for nonbleeding patients in unstable condition (such as those with fever or infection), platelet transfusion is recommended if the platelet count is < 20 × 10^9^/L. For patients undergoing invasive procedures and surgical preventive transfusion, platelet transfusion is recommended if the platelet count is < 20 × 10^9^/L in patients with an indwelling central venous catheter. For patients undergoing ECLS, elective diagnostic lumbar puncture, or non-axonal surgery, platelet transfusion is recommended if the platelet count is < 50 × 10^9^/L. For patients with scheduled spinal anesthesia, platelet transfusion is recommended if the platelet count is ≤80 × 10^9^/L. For patients with scheduled neurosurgery or eye surgery, platelet transfusion is recommended if the platelet count is ≤100 × 10^9^/L. For patients with active bleeding, the platelet count should be maintained at 50 × 10^9^/L. For patients undergoing cardiothoracic surgery with coagulation abnormalities and major microvascular bleeding, platelet transfusion is recommended if the platelet count is < 100 × 10^9/^L. [157]

The platelet transfusion amount should be individualized, taking into account the patient’s weight, spleen function, and other depletion factors. The dose is generally 1 unit of apheresis platelets or an equivalent dose of platelet concentrate per dosing; ≥ 2 units of apheresis platelets may be transfused in the case of severe life-threatening bleeding. After transfusion, the dose should be adjusted based on its effectiveness; the goal is to transfuse the minimum dose required to maintain the platelet count target. One unit of platelets can theoretically increase the platelet count by 4 × 10^9^ to 8 × 10^9^/L in adults (70 kg body weight).

For patients receiving antiplatelet therapy, bleeding-induced thrombocytopenia significantly increases mortality, blood loss, and the surgery rate [160, 161]. For patients receiving antiplatelet therapy for platelet dysfunction, their prognosis is related to the antiplatelet drugs given. Studies have shown that for patients taking aspirin, severe trauma and even brain injury do not increase mortality but will require significantly more transfused blood [162]. For patients taking clopidogrel, severe trauma will significantly increase mortality [163, 164]. Therefore, for patients undergoing antiplatelet therapy, platelet transfusion is recommended in cases of persistent bleeding with platelet dysfunction or even thrombocytopenia [165]. For patients receiving antiplatelet therapy, platelet transfusion is recommended in cases of surgical treatment for cerebral hemorrhage [166]. However, platelet transfusion is not recommended for patients receiving antiplatelet therapy who have cerebral hemorrhage that does not require surgical treatment [167, 168]. The use of platelet count as an indication for platelet transfusions has not been shown to improve the outcomes of critical care patients [169, 170]. The use of platelet function parameters to guide platelet transfusion in critical care patients with thrombocytopenia may be beneficial for improving the outcomes of these patients [171–173].

#### Recommendation 23: ABO/RhD-compatible platelets are preferred for blood transfusion

ABO blood group selection for platelet transfusion: ABO-compatible platelets are preferred forplatelet transfusion; ABO-incompatible platelets (i.e., the second best choice) may be used in cases of life-threatening bleeding with no available ABO-compatible platelets [174]. Platelet transfusion with minor ABO incompatibility (the presence of antibodies in donor plasma against the red blood cell and platelet ABH antigens of the recipient) and platelet transfusion with major ABO incompatibility (the presence of antibodies in recipient plasma against red blood cell and platelet ABH antigens of the donor) pose the risk of hemolytic reaction. Generally, adult patients are able to dilute or neutralize incompatible antibodies in the transfused plasma, and most show a positive result in direct antiglobulin tests, with no signs of hemolytic reaction [175]. Foreign studies have shown that ABO incompatibility has no significant effect on the effectiveness of transfusion. Therefore, some researchers suggest that the effect of ABO incompatibility on the effectiveness of platelet transfusion is negligible in patients who do not require long-term platelet transfusion, especially surgical patients [176].

RhD blood group selection for platelet transfusion: D antigen is the most immunogenic antigen in the Rh system. The Rh blood group is either “Rh positive” or “Rh negative” based on the presence of D antigen in red blood cells. Despite a lack of D antigen in platelets, platelet products contain a certain amount of red blood cells (< 1 mL of red blood cells per therapeutic unit of apheresis platelets, and more in platelet concentrate), which can cause sensitization and thereby pose a risk to the safety of future blood transfusions in RhD-negative patients and a risk of neonatal hemolytic disease during future pregnancies in female patients. Therefore, RhD-negative patients should receive RhD-negative platelets whenever possible, but RhD-incompatible platelets may be transfused in emergencies [174]. Anti-D antibodies are recommended in RhD-negative women with child-bearing potential who have received RhD-positive platelet transfusion [158].

#### Recommendation 24: Effectiveness should be evaluated after platelet transfusion

Platelet transfusion refractoriness (PTR) refers to alack of a significant platelet increase or even a decline 2 consecutive times after adequate platelet transfusion, with no improvement in clinical bleeding [177]. PTR may be caused by nonimmune or immune factors. Common nonimmune factors include traumatic coagulopathy, splenomegaly, DIC, and excessive platelet consumption due to inappropriate transfusion. The following precautions are recommended for platelet transfusion: mix the blood bag gently before transfusion and transfuse as fast as possible (per patient tolerance); unused platelets should not be stored in the refrigerator but can be kept at room temperature for a short time, preferably on a platelet shaker. Alloimmune PTR, the most common immune factor-induced disease, is defined as a platelet increase < 5 × 10^9^/L in 10 min to 1 h, 2 consecutive times, after the transfusion of ABO-compatible platelets in patients with thrombocytopenia due to loss of bone marrow function who have no clear nonimmunological factors [178]. Alloimmune PTR is caused by the production of antiplatelet antibodies in response to repeated platelet transfusions and subsequent immune responses with newly transfused platelets, which causes the destruction of transfused platelets and PTR [179]. Antigen A, antigen B, human leukocyte antigen (HLA) antibodies, and human platelet antigen (HPA) are common causes of immune PTR. Therefore, PTR patients should receive ABO-, HLA-, or HPA-compatible platelets whenever possible [180, 181].

### Plasma exchange

#### Recommendation 25: Plasma exchange eliminates abnormal immune antibodies and reduces platelet destruction

Plasma exchange is effective for platelet destruction due to abnormal immune antibodies and is recommended for TTP, CAPS, refractory ITP, atypical HUS, HELLP, and PSP. It is not recommended for Shiga toxin-associated classic HUS with no central nervous system symptoms or for *Streptococcus pneumoniae*-associated HUS [182, 183].

Plasma exchange can be used as conventional treatment or an intensive treatment. For TTP, the recommended conventional treatment is 40 to 60 mL/kg per session, ≤ 2 times per day, until the symptoms have improved, LDH is normal, and platelet count is normal or elevated [184]. If LDH remains elevated or platelet count is persistently low after 7 days of daily plasma exchange, refractory TTP is considered; this condition requires intensive plasma exchange (2 times per day) combined with glucocorticoids or immunosuppressive agents [142, 185, 186].

Plasma exchange may be performed in patients undergoing ECMO who have heparin-induced HIT or significant thrombosis and require the rapid elimination of excessive anti-PF4-heparin antibodies [187, 188]. Several studies have shown that plasma exchange is effective for HIT [189].

For patients with septic shock, the incidence of thrombocytopenia may be 50% or higher, with a significantly increased risk of mortality [190, 191]. Studies have shown that early plasma exchange in patients with septic shock, especially in patients who require high-dose vasoactive drugs to maintain hemodynamics, reduces the dose of vasoactive drugs, removes inflammatory cytokines, and reduces capillary leakage and platelet consumption [192–194].

### Surgical treatment

#### Recommendation 26: Splenectomy should be used with caution for thrombocytopenia

Splenectomy eliminates the site responsible for the production of autoantibodies and the destruction of red blood cells and platelets. It can be used to treat immune antibody-mediated thrombocytopenia refractory to conventional treatment, such as refractory ITP, TTP, and AIHA [89, 142, 195]. Splenectomy is indicated in patients whose course of disease is > 6 months and who do not respond to regular steroid treatment; patients who respond to steroid treatment but require high-dose maintenance therapy; and patients contraindicated for steroid therapy [89]. The main complications of surgical treatment are bleeding and secondary infection. Laparoscopic minimally invasive surgery is an effective method for reducing complications [196].

### Prevention

#### Recommendation 27: Influencing factors of thrombocytopenia need to be actively controlled during ECLS

ECLS refers to the use of extracorporeal equipment to completely or partially replace organ function to provide life support in the case of life-threatening organ dysfunction. ECLS broadly includes ECMO, which supports cardiopulmonary function; RRT, which supports renal function; and ALSS, which supports liver function [197].

During ECLS, blood passes through the extracorporeal circulation line. The contact between blood and circuit material may activate the extrinsic coagulation pathway and initiate coagulation, resulting in the additional consumption of platelets and coagulation factors [198]. Anticoagulant drugs that prevent coagulation in the circuit may also affect the patient’s coagulation state, increasing the risk of bleeding-induced thrombocytopenia or inducing HIT [199]. Guru et al. [200] showed that among critical care patients scheduled to receive continuous RRT (CRRT), 65% have thrombocytopenia, and an additional 20% have thrombocytopenia during CRRT. Patients receiving CRRT and heparin anticoagulation have more significant platelet decline than patients receiving heparin anticoagulation alone, without CRRT. The 4Ts score indicates that most patients meet the criteria for a diagnosis of HIT; however, the rate of markedly positive antibodies is actually low [201]. Choi et al. [202] showed that the incidence of thrombocytopenia is up to 83% in patients receiving ECMO. VA-ECMO is more likely than VV-ECMO to cause thrombocytopenia. This is related to platelet consumption due to athrombus caused by membrane oxygenator-induced vWF aggregation [203]. The duration of ECMO is largely unrelated to thrombocytopenia [204]. During ECMO, the incidence of HIT is approximately 20%. In most cases, PF4 antibody results are positive during ECMO.HIT should be considered in cases of frequent abnormalities in the ECMO circuit, progressive platelet decline, and high levels of PF4-specific IgG antibodies [205]. Therefore, during ECLS, clinicians should comprehensively evaluate coagulation function, choose an appropriate anticoagulation regimen, and make every effort to control influencing factors of thrombocytopenia. In cases of thrombocytopenia during ECLS, clinicians should actively identify the cause and discontinue anticoagulation therapy if needed [83, 206].

## Data Availability

Not applicable.

## Abbreviations

AA: Aplastic anemia
ACS: Acute coronary syndrome
AFLP: Acute fatty liver of pregnancy
AIHA: Autoimmune hemolytic anemia
ALSS: Artificial liver support system
ALT: Alanine aminotransferase
AML: Acute myeloid leukemia
ANA: Antinuclear antibodies
APS: Antiphospholipid syndrome
APTT: Activated partial thromboplastin time
AST: Aspartate aminotransferase
CAPS: Catastrophic antiphospholipid syndrome
CMV: Cytomegalovirus
CRP: C-reactive protein
DD: D-dimer
DIC: Disseminated intravascular coagulation
DNA: Deoxyribonucleic acid
ECLS: Extracorporeal life support
ECMO: Extracorporeal membrane oxygenation
EDTA: Ethylenediaminetetraacetic acid
EHS: Exertional heat stroke
FDP: Fibrin degradation products
HBV: Hepatitis B virus
HCV: Hepatitis C virus
HELLP: Hemolysis, elevated liver enzymes, and low platelets syndrome
HIT: Heparin-induced thrombocytopenia
HIV: Human immunodeficiency virus
HUS: Hemolytic uremic syndrome
HLA: Human leukocyte antigen
HLH: Hemophagocytic lymphohistiocytosis
HPA: Human platelet antigen
IABP: Intra-aortic balloon counter pulsation
ICU: Intensive care unit
IL: Interleukin
IVIg: Intravenous immunoglobulin
ITP: immune thrombocytopenia
LAC: Lupus anticoagulant
LDH: Lactic acid dehydrogenase
LTA: Light transmission aggregometry
MDS: Myelodysplastic syndrome
NETs: Neutrophil extracellular traps
NOAC: Novel oral anticoagulants
PAI: Plasminogen activator inhibitior
PCT: Procalcitonin
PF: Platelet factor
PNH: Paroxysmal nocturnal hemoglobinuria
PT: Prothrombin time
PTP: Posttransfusion purpura
PTR: Platelet transfusion refractoriness
RA: Rheumatoid arthritis
RCT: Randomized controlled trial
RF: Rheumatoid factor
RRT: Renal replacement therapy
SLE: Systemic lupus erythenlatosus
TFPI: Tissue factor pathway inhibitor
TM: Thrombomodulin
TMA: Thrombotic microangiopathy
t-PA: Tissue-type plasminogen activator
TPO: Thrombopoietin
TT: Thrombin time
TTP: Thrombocytopenic purpura
VAD: Ventricular assist device
VWD: Von Willebrand disease
